# Association between the feeling of vocal comfort and social security when wearing a protective mask: a study with transgender people

**DOI:** 10.1590/2317-1782/e20250105en

**Published:** 2026-04-10

**Authors:** Isabella Marins, Kelly da Silva, Aline Wolf, Juliana Portas, Julie Vigano, Rodrigo Dornelas

**Affiliations:** 1 Departamento de Fonoaudiologia, Faculdade de Medicina, Universidade Federal do Rio de Janeiro – UFRJ - Rio de Janeiro (RJ), Brasil.; 2 Universidade Federal de Sergipe – UFS - Lagarto (SE), Brasil.; 3 Universidade de São Paulo – USP - Ribeirão Preto (SP), Brasil.; 4 Centro de Estudo da Voz – CEV - São Paulo (SP), Brasil.; 5 Universidade Federal de Santa Catarina – UFSC - Florianópolis (SC), Brasil.

**Keywords:** Transgender People, Quality of Life, Voice Quality, Speech, Language and Hearing Sciences, COVID-19, Pandemic

## Abstract

**Purpose:**

To analyze the influence of facial mask usage on vocal production and social interaction in transgender individuals.

**Methods:**

A cross-sectional study employing a convenience sample recruited via the snowball method was conducted. Data were collected through an online questionnaire comprising questions about gender identification, comfort with mask usage, and perceived social security, in addition to the validated instruments Vocal Disadvantage Index-10 (IDV-10) and Vocal Fatigue Index (IFV). Descriptive statistics and logistic regression (with a significance level of 5%) were used to identify predictors of social security perception.

**Results:**

A total of 85 transgender individuals participated, with a mean age of 30.4 years, including 44.7% trans men and 55.3% trans women. Although only 43.5% of participants reported feeling comfortable with mask use, 75.3% experienced an increased sense of social security. Significant correlations were found between the IDV-10 and IFV scores, and logistic regression analysis revealed that comfort with mask usage significantly predicted the perception of social security (Z = -3.11; p = 0.002).

**Conclusion:**

Facial mask usage plays a dual role in the transgender experience, inducing vocal discomfort while enhancing feelings of social security.

## INTRODUCTION

Transsexuality is a person’s non-identification with the gender assigned at birth^(1)^. Transgender people have a strong sense of their gender and reaffirm this feeling by adopting a new name or modifying their bodies^(2)^. Thus, there is a social mobilization in search of legitimacy, social respect, and identity recognition.

The human voice, in turn, is a central element in communication and identity construction, being especially relevant for transgender people in their gender affirmation. It allows the externalization of personality and emotions and reflects cultural and environmental aspects. The voice, therefore, is an essential part of personal identity and can be an important marker in confronting situations of discrimination, such as transphobia.

The mandatory use of face masks amid the COVID-19 pandemic brought new challenges to communicative interaction between people. Masks can affect vocal production by muffling sound, reducing sound pressure, and increasing vocal effort, contributing to vocal fatigue and possible phonotrauma^(3)^. The literature has already discussed these effects, but in general terms and often focused on the cisgender population.

Contrarily, studies investigating the effects of face mask use on the voice and socialization of transgender people are still scarce. Considering the centrality of the voice in affirming gender identity and the frequent exposure of this group to situations of discrimination, mask use may plausibly have a particular impact on this population regarding both vocal production and psychosocial safety in social interactions.

It should be clarified that this study did not focus on mask use in the general population. The purpose is specifically on transgender people and the vocal and psychosocial effects that the use of face masks can have on this group. Thus, the mask is considered an element that interacts with identity, vocal, and social variables specific to transgender experiences and can influence both vocal self-perception and safety and comfort in social interactions.

This study aimed to analyze the influence of the use of face masks on vocal production and socialization of transgender people. The relevance of this investigation lies in the intersection of social, psychological, and physical factors that shape the vocal experience and social circulation of transgender people, especially in contexts where the visibility and expressiveness of the voice play a central role in identity affirmation. By focusing specifically on the effects caused by masks on transgender people, we seek to contribute to the understanding of how this protective resource impacts fundamental dimensions of their daily lives.

## METHODS

This study rigorously followed ethical guidelines and was approved by the Research Ethics Committee of the Federal University of Rio de Janeiro under approval number 5.344.837 and CAAE 54150321.1.0000.5261. This is a cross-sectional observational study with a convenience sample, using snowball sampling for data collection, in which initial participants indicate new participants until the desired number is reached^(4)^. The researcher invited the first participants by email, having chosen them because they were active in social movements in Brazil.

Participants included transgender people of any gender, aged 18 or older, excluding those over 60 years old or who did not complete the questionnaires. Data were collected remotely, using a form on Google Forms.

The researchers used a brief questionnaire they had developed, consisting of three questions about comfort in using the mask ("Do you feel comfortable using face masks?"), perception of vocal changes during the period of use of this protection ("Did you experience any vocal changes with the use of face masks during the pandemic?"), and social safety when using this facial protection ("Did you feel safer/more socially secure with the use of face masks?").

In addition to the questionnaire, the study used the Voice Handicap Index-10 (VHI-10)^(5)^ and the Vocal Fatigue Index (VFI)^(6)^. The VHI-10 consists of 10 questions and is a validated instrument for assessing a subject's perception of their voice handicap. The result produces a single total score obtained by summing the responses to the items, ranging from 0 to 40 points. Each item uses a 5-point Likert-type scale, where 0 means "never", and 4 means "always"; the higher the score, the greater the perception of voice handicap^(5)^.

VFI is a validated 19-item questionnaire that aims to identify and measure vocal fatigue. It is organized into three domains: Fatigue and vocal limitation, Physical discomfort associated with voice, and Recovery with vocal rest. In the first two domains, higher scores indicate greater fatigue and discomfort, while in the third domain, they indicate greater improvement in symptoms. Each question uses a Likert-type scale ranging from 0 to 4; the score is obtained by simply summing the responses. The total score ranges from 0 to 76, with specific subscales for Fatigue and vocal limitation (0 to 44), Physical discomfort associated with voice (0 to 20), and Recovery with vocal rest (0 to 12)^(6)^.

Statistical analysis was conducted using binomial regression. The dependent variable was the responses to the question, "Did you feel safer/more socially secure with the use of face masks?". All other independent variables (age, gender, VHI-10 and VFI results, and responses to the questions, "Do you feel comfortable using face masks?" and "Did you experience any vocal changes during the pandemic?") were tested, and the final model consisted only of the significant independent variable.

Spearman's correlation analysis was also performed between the total VHI-10 score (total points) and the scores of the VFI domains (Fatigue and limitation, Physical discomfort associated with voice, and Recovery with vocal rest). This analysis aimed to verify possible associations between self-reported vocal impact and factors related to the use of face masks. The significance level was set at 5% (p < 0.05). All analyses were performed using JAMOVI software, version 2.3.

## RESULTS

The study included 85 transgender people, with a mean age of 30.4 ± 9.31 years, of which 38 (44.7%) were transgender men, and 47 (55.3%) were transgender women. Table 1 presents their ages ​​and VHI-10 and VFI scores by gender.

**Table 1:** Age and VHI-10 and VFI scores according to gender

	**Gender**	**Mean**	**Median**	**Standard deviation**	**Minimum**	**Maximum**
Age	Transgender men	28.0	27.0	7.54	19	54
	Transgender women	32.3	32.0	10.19	18	57
Total VHI-10	Transgender men	12.2	11.5	7.81	0	27
	Transgender women	14.5	14.0	8.29	0	37
VFI - Fatigue and Vocal Limitation	Transgender men	13.9	13.5	7.74	0	30
	Transgender women	16.4	15.0	9.62	0	35
VFI - Physical discomfort associated with voice	Transgender men	3.87	2.00	4.97	0	20
	Transgender women	3.04	1.00	3.80	0	14
VFI - Recovery with vocal rest	Transgender men	6.18	8.00	4.42	0	12
	Transgender women	6.87	8.00	4.56	0	12
VFI - Total	Transgender men	23.9	24.0	12.6	4	54
	Transgender women	26.3	28.0	14.0	0	47

Descriptive statistical analysis

Caption: VHI-10 = Voice Handicap Index 10; VFI = Vocal Fatigue Index

As shown in Table 2, 56.5% of participants did not feel comfortable using protective masks, while 43.5% reported comfort; 65.9% stated that they had not noticed any vocal changes during the pandemic, while 34.1% reported changes; and finally, 75.3% felt safer socially with the use of the mask, compared to 24.7% who did not report this feeling. Correlation analysis indicated significant relationships between VHI-10 scores and VFI domains, except for the "Recovery with vocal rest" domain (ρ = 0.03; p = 0.744).

**Table 2:** Measures of fit and coefficients of the model

					**Testing the Global Model**		
**Model**	**Deviance**	**AIC**	**R^2^_CS_**	**R^2^_CS_**	**χ^2^**	**df**	**p**
1	80.0	84.0	0.158	0.241	15.0	1	< .001			
Model coefficients – “Did you feel safer/more socially secure with the use of face masks?”							
**Predictor**				**Estimates**	**Standard error**	**Z**	**p**
Intercept				-0.423	0.295	-1.43	0.152
Do you feel comfortable using face masks?							
Yes – No				-2.439	0.785	-3.11	0.002

Note. The estimates represent the log odds of "Did you feel safer/more socially secure with the use of face masks?” = “No" vs. "Did you feel safer/more socially secure with the use of face masks?” = “Yes"

Binomial logistic regression

Caption: AIC = Akaike information criterion; R^2^_CS_ = Cox and Snell coefficients of determination; χ^2^ = chi-square test; df = degrees of freedom

Binomial logistic regression was used to investigate the predictors of "Did you feel safer/more socially secure with the use of face masks?". The fitted model was significant with adjusted Cox & Snell R^2^ values ​​of 0.158 and Nagelkerke R^2^ values ​​of 0.241, suggesting that a substantial part of the variability was explained by the predictor variable: "Did you feel safer/more socially secure with the use of face masks?" was statistically significant (Z = -3.11; p = 0.002).

Collinearity checks were performed, which indicated ideal values ​​(VFI = 1.00; Tolerance = 1.00), ensuring the robustness of the model. The correlation analysis between the total VHI-10 score and the VFI domain scores demonstrated distinct associations between these constructs. A moderate and statistically significant positive correlation was observed between the VHI-10 and the Fatigue and vocal limitation VFI domain (Spearman's rho = 0.530; p < 0.001), indicating that higher voice handicap levels are associated with a greater perception of fatigue and limitation in voice use. Similarly, a weak but significant positive correlation was identified between the VHI-10 and the Physical discomfort associated with voice (rho = 0.333; p = 0.002), suggesting that greater voice handicap is also related to a greater presence of vocal physical symptoms.

On the other hand, no statistically significant correlation was observed between the total VHI-10 score and Recovery with vocal rest (rho = 0.034; p = 0.759), indicating that the perception of voice handicap is not associated, in this group, with the ability to recover vocally after periods of rest. These findings reinforce the convergence between measures of vocal impairment and vocal fatigue, especially in aspects related to functional limitation and physical discomfort.

## DISCUSSION

This study sought to understand, through the vocal self-perception of transgender people, how the use of face masks influenced vocal use and social interaction during the COVID-19 pandemic. According to the participants' reports, the mask reduced voice-related anxiety by attenuating characteristics that could be perceived as discrepant with gender identity. Thus, the reported comfort refers not only to physical protection against the virus but also to a positive effect on the social perception of gender identity.

The voice is an important identification and communication trait. It is important to consider how the voice of transgender people is qualified to understand their needs regarding physical and psychosocial well-being. Factors such as social, psychological, and physical well-being influence people's quality of life^(7)^ and, consequently, vocal self-perception.

Given the stigma experienced by transgender people, leading to the intolerance they suffer, known as transphobia, quality of life is a relevant aspect to discuss. Transphobia directly impacts people's quality of life, contributing to illness.

Health actions must be developed for transgender people to improve their quality of life, based on knowledge of specific needs^(7)^. This study aimed to understand voice-related complaints and their relationship with mask use during the COVID-19 pandemic.

The SARS-CoV-2 pandemic required the use of face masks, which, in turn, interfered with the use of the voice of the entire population and, specifically, the voice of transgender people. The data analysis revealed that, although some people felt uncomfortable wearing masks, most reported a feeling of greater social security. This discrepancy suggests that, even in the face of physical discomfort or vocal changes, a perceived benefit in terms of protection against discrimination may prevail. Logistic regression results reinforce this interpretation, demonstrating that comfort with the mask is a significant predictor of feelings of social safety.

As previous studies pointed out, the voice is a central element in the construction of identity and social communication^(7,8)^. In the context of transgender people, mask use can function as a resource to mitigate vocal aspects that, in certain situations, may be a source of discomfort or stigma. This significant percentage corroborates that protective masks promote social security, since they conceal part of the face, modify the sound of the voice, and protect the person from transphobic actions. This number indicates the mask as support in protecting identity, considering the strong discrimination that affects this population^(9)^.

In the context of vocal self-perception, the mask plays an ambiguous role. On the one hand, it acts as a psychosocial defense mechanism, favoring what is called gender passing. This concept refers to how a person is perceived in accordance with their gender identity and is associated with comfort, social security, and gender expression^(8)^, reducing exposure to situations of discrimination^(9)^. On the other hand, it can cause sound muffling, as demonstrated by a reduction ​​of up to 3 and 4 dB with common masks and up to 12 dB with N95 masks^(10)^, which implies greater vocal effort and potential fatigue.

Transgender people seek a voice that matches their gender identity, and the voice is often one of the aspects that most interferes with the process of passing^(11)^. This duality reinforces the idea that, for transgender people, the use of a mask goes beyond biological protection and is configured as a tool for identity affirmation and security.

In this study, the values ​​corresponding to vocal fatigue were similar between transgender men and women. The opposite situation was found in a study that found that cisgender women, because they use their voice more, self-perceive greater vocal fatigue than cisgender men^(12)^. Comparative studies in cisgender populations demonstrate that women may report greater voice handicap in some contexts. However, this study found no significant differences between transgender men and transgender women. The transition processes experienced by transgender men and women have similarities, including social, hormonal, and, in some cases, surgical dimensions. Although these aspects may involve specific and distinct objectives, they share common elements that contribute to the convergence of transition pathways, possibly due to psychosocial needs and stress involved in these experiences.

Analysis of the scales demonstrated significant correlations between VHI-10 and two VFI domains, with the exception of the "Recovery with vocal rest". This finding indicates that increased perception of voice handicap is associated with higher levels of fatigue and physical discomfort, corroborating the need for a speech-language-hearing approach that considers both physical and psychosocial aspects in intervention with this population.

Furthermore, the data indicated no significant differences in the protocol scores (VHI-10 and VFI) between transgender men and women, which may reflect the common processes of social transition, and, in some cases, hormonal and surgical transition experienced by these groups^(7)^. This finding suggests that, despite possible individual variations, the transgender population has homogeneous needs and challenges related to vocal production, emphasizing the need for inclusive and specific intervention strategies. This result is intriguing, since another study with cisgender men and women likewise found no statistical association between VFI and gender^(11)^. Additionally, investigations with cisgender populations suggest that, although VFI is effective regardless of gender, they may perceive vocal problems differently, which was not observed in the present study.

The data highlight the urgency of speech-language-hearing actions that address both technical and psychosocial aspects of vocal production, helping to improve quality of life and formulate inclusive public policies. The negative aspects in vocal production pointed out in the results suggest the need for speech-language-hearing care accessible to the population in accordance with the principles of universality and integrality of the Brazilian Unified Health System (SUS), which is not yet happening^(13)^. The relevance of this study is reinforced by the fact that, although the SUS recognizes the importance of speech-language-hearing pathologists in the multidisciplinary healthcare team for transgender people, such professionals are not yet included in the Transsexualization Process Ordinance of the Ministry of Health^(3)^.

Given that vocal quality is directly linked to quality of life, greater emphasis on studies such as this, involving the vocal self-perception of transgender people and the COVID-19 pandemic, may serve as an important reference in updating or developing new public policies for this population^(7)^.

Further research is warranted, incorporating more specific exclusion criteria, such as delimiting voice professionals, and adopting longitudinal designs to more robustly elucidate the long-term effects of mask use on vocal self-perception and social security of transgender people. This broader perspective can contribute to the formulation of more effective preventive and intervention strategies, strengthening the role of speech-language-hearing pathologists in the context of comprehensive healthcare for this population.

This study has limitations, such as the use of a convenience sample, which may limit the generalizability of the results. However, this type of sample is justified by the fact that the target population, transgender people, suffer invisibility in population censuses, which also justifies the way the sample was conducted. To minimize this limitation, the study sought well-defined inclusion criteria and a detailed characterization of the participants to help contextualize the findings.

Another limitation concerns the lack of a control group, which could allow for direct comparisons between transgender and cisgender people regarding the effects of mask use. However, this methodological choice aligns with the study's main objective: to understand the vocal and social experiences of transgender people in the context of wearing face masks. The aim is not to establish contrasts with other groups, but to give visibility to the specific experiences of this population, whose reality is marked by its own identity, social, and communicative intersections.

## CONCLUSION

The study identified that the use of face masks plays a dual role in the vocal and social experience of transgender people. Despite the discomfort reported by the participants, the feeling of social safety was significantly elevated, indicating that the psychosocial benefits can outweigh the physical challenges associated with prolonged mask use. The results revealed that comfort with mask use is a relevant predictor of this feeling of safety, highlighting the importance of this factor in the daily lives of the study population.

Moreover, the correlations between VHI-10 scores and VFI domains suggest that the perception of voice handicap is associated with increased vocal fatigue and physical discomfort. The absence of statistically significant differences between transgender men and women in the applied protocols points to a possible homogenization of vocal experiences resulting from social, hormonal, and/or surgical transition processes.

These findings highlight the urgency of developing speech-language-hearing intervention strategies that integrate technical aspects of vocal production with psychosocial dimensions, promoting greater comfort, vocal authenticity, and well-being. The study also points to the need to expand research with more diverse and longitudinal samples and to implement public policies that consider the specific needs of transgender people in accessing vocal health, safe socialization, and the recognition of their identities in diverse social contexts.
