# Supplementary material for: Association between the feeling of vocal comfort and social security when wearing a protective mask: a study with transgender people
Source: Codas. 2026 Apr 10;38(2):e20250105. doi: 10.1590/2317-1782/e20250105en (PMC13075830; doi:10.1590/2317-1782/e20250105en)
Supplement: Translation

# Translation: Associação entre a sensação de conforto vocal e de segurança social no uso de máscara de proteção: um estudo com pessoas trans

**DOI:** 10.1590/2317-1782/e20250105pt

**Authors:** Isabella Marins, Kelly da Silva, Aline Wolf, Juliana Portas, Julie Vigano, Rodrigo Dornelas

**Affiliations:** 1 Departamento de Fonoaudiologia, Faculdade de Medicina, Universidade Federal do Rio de Janeiro – UFRJ - Rio de Janeiro (RJ), Brasil.; 2 Universidade Federal de Sergipe – UFS - Lagarto (SE), Brasil.; 3 Universidade de São Paulo – USP - Ribeirão Preto (SP), Brasil.; 4 Centro de Estudo da Voz – CEV - São Paulo (SP), Brasil.; 5 Universidade Federal de Santa Catarina – UFSC - Florianópolis (SC), Brasil.

**Keywords:** Pessoas Transgênero, Qualidade de Vida, Qualidade da Voz, Fonoaudiologia, COVID-19, Pandemia

## Abstract

**Objetivo:**

Analisar a influência do uso de máscara de proteção facial na produção vocal e na socialização de pessoas trans.

**Método:**

Estudo transversal, de amostra por conveniência, realizado com pessoas trans. Para a coleta de dados foi utilizado um breve questionário composto por três questões versando sobre identidade de gênero, conforto no uso da máscara e segurança social ao utilizar esta proteção. O instrumento validado utilizado foi o Índice de Desvantagem Vocal: 10 (IDV-10) e o Índice de Fadiga Vocal (IFV). A coleta foi realizada durante a pandemia de COVID-19 por ter sido o período com maior uso de máscara facial registrado. A análise estatística foi conduzida por meio de estatística descritiva (medidas de tendência central e de dispersão) e inferencial, por meio da regressão logística (nível de significância de 5%).

**Resultados:**

Participaram 85 pessoas trans, com idade média de 30,4 anos, sendo 44,7% homens trans e 55,3% mulheres trans. Embora apenas 43,5% dos participantes tenham relatado sentir conforto com o uso da máscara, 75,3% afirmaram sentir maior segurança social durante seu uso. Os escores do IDV-10 e do IFV apresentaram correlações significativas, e a regressão logística demonstrou que o conforto com o uso da máscara foi um preditor estatisticamente significativo para a sensação de segurança social (Z = -3,11; p = 0,002).

**Conclusão:**

O uso de máscara de proteção facial exerce um duplo papel na experiência de pessoas trans, causando desconforto vocal, mas promovendo uma sensação ampliada de segurança social.

## INTRODUÇÃO

A transexualidade é uma condição de não identificação da pessoa com o gênero designado ao nascimento^(1)^. As pessoas transexuais têm um sentimento sólido de seu gênero, e adotar um novo nome ou modificar seus corpos são formas de reafirmar esse sentimento^(2)^. Nesse contexto, observa-se uma mobilização social em busca de legitimidade, respeito social e reconhecimento da identidade.

A voz humana, por sua vez, é um elemento central na comunicação e na construção da identidade, sendo especialmente relevante para pessoas trans em processos de afirmação de gênero. Ela permite a exteriorização da personalidade, das emoções, e reflete aspectos culturais e ambientais. A voz, portanto, torna-se parte essencial da identidade pessoal, podendo ser um marcador importante no enfrentamento de situações de discriminação, como a transfobia.

Em meio à pandemia de COVID-19, o uso obrigatório de máscaras de proteção facial trouxe novos desafios para a interação comunicativa entre as pessoas. As máscaras podem afetar a produção vocal ao gerar abafamento do som, reduzir a pressão sonora e aumentar o esforço vocal, contribuindo para fadiga vocal e possíveis fonotraumas^(3)^. Esses efeitos já vêm sendo discutidos na literatura, mas de forma geral e, muitas vezes, voltados à população cisgênera.

Entretanto, ainda são escassos os estudos que investiguem os efeitos do uso de máscaras de proteção facial sobre a voz e a socialização de pessoas trans. Considerando a centralidade da voz na afirmação da identidade de gênero e a exposição frequente desse grupo a situações de discriminação, é plausível que o uso de máscaras exerça um impacto particular nesta população, tanto do ponto de vista da produção vocal quanto da segurança psicossocial nas interações sociais.

Cabe esclarecer, que este estudo não teve como foco o uso de máscaras na população geral. O propósito está especificamente nas pessoas trans e nos efeitos vocais e psicossociais que o uso de máscaras de proteção facial pode ter nesse grupo. Assim, a máscara é considerada um elemento que interage com variáveis identitárias, vocais e sociais próprias das vivências trans, podendo influenciar tanto na autopercepção vocal quanto na segurança e no conforto nas interações sociais.

Este estudo tem como objetivo analisar a influência do uso de máscaras de proteção facial na produção vocal e na socialização de pessoas trans. A relevância desta investigação está na interseção de fatores sociais, psicológicos e físicos que moldam a experiência vocal e a circulação social de pessoas trans, especialmente em contextos em que a visibilidade e a expressividade da voz desempenham papel central na afirmação identitária. Ao focar especificamente nos efeitos provocados pelas máscaras em pessoas trans, busca-se contribuir para a compreensão de como esse recurso de proteção impacta dimensões fundamentais do cotidiano desse grupo.

## MÉTODOS

Este estudo seguiu rigorosamente os preceitos éticos e foi aprovado pelo Comitê de Ética em Pesquisa da Universidade Federal do Rio de Janeiro sob o parecer de n° 5.344.837 e o CAAE 54150321.1.0000.5261. Trata-se de um estudo observacional do tipo transversal, com amostra por conveniência, utilizando a técnica de amostragem *Snowball* para a coleta de dados, na qual participantes iniciais indicam novos participantes até alcançar o número desejado^(4)^. Os primeiros participantes foram convidados por e-mail pela pesquisadora e foram escolhidos por serem pessoas atuantes nos movimentos sociais do Brasil.

Participaram pessoas trans, de qualquer gênero, com idade superior ou igual aos 18 anos, sendo excluídas aquelas com idade superior a 60 anos ou que não finalizaram o preenchimento dos questionários. A coleta de dados ocorreu de forma remota, por meio de um formulário na plataforma *Google Forms*.

Foi utilizado um breve questionário idealizado pelos pesquisadores composto por três questões sobre conforto no uso da máscara (“Você se sente confortável com o uso das máscaras de proteção facial?”), percepção sobre alteração vocal causada durante o período de uso desta proteção (“Você sentiu alguma alteração vocal neste período de pandemia com o uso de máscaras de proteção facial?”) e segurança social ao utilizar esta proteção facial (“Você se sentiu mais segura/seguro socialmente com o uso de máscaras de proteção facial?”).

Além do questionário, foram utilizados o Índice de Desvantagem Vocal- 10 (IDV-10)^(5)^ e o Índice de Fadiga Vocal (IFV)^(6)^. O IDV-10 é composto por dez questões, sendo um instrumento validado para avaliar a percepção do sujeito com relação à sua desvantagem vocal. O resultado produz um escore total único que é obtido somando as respostas dos itens, variando de 0 a 40 pontos; cada item utiliza uma escala do tipo Likert de 5 pontos, em que 0 significa “nunca” e 4 significa “sempre”; quanto maior o escore, maior a percepção de desvantagem vocal^(5)^.

O IFV é um questionário validado, composto por 19 questões, que tem por objetivo identificar e mensurar a fadiga vocal. É organizado em três domínios: Fadiga e restrição vocal; Desconforto físico associado à voz; e Recuperação com repouso vocal. Nos dois primeiros domínios, escores mais altos indicam maior fadiga e desconforto, enquanto no terceiro domínio indicam maior melhora nos sintomas. Cada questão usa uma escala do tipo Likert com variação de 0 a 4, o escore é obtido por meio da soma simples das respostas. O escore total varia de 0 a 76, com subescalas específicas para fadiga e restrição vocal (0 a 44), desconforto físico associado à voz (0 a 20) e recuperação com repouso vocal (0 a 12)^(6)^.

A análise estatística foi conduzida por meio de regressão binomial sendo a variável dependente as respostas da pergunta “Você se sentiu mais segura/seguro socialmente com o uso de máscaras de proteção facial?”. Todas as demais variáveis independentes (idade, gênero, resultados do IDV-10 e do IFV e as respostas às perguntas “Você se sente confortável com o uso das máscaras de proteção facial?” e “Você sentiu alguma alteração vocal neste período de pandemia?”) foram testadas e o modelo final foi constituído apenas pela variável independente significativa.

Ainda, foi realizada a análise de correlação de Spearman entre o escore total do IDV-10 (total de pontos) e os escores dos domínios do IFV (Fadiga e restrição; Desconforto físico associado à voz e Recuperação com repouso vocal). Essa análise teve como objetivo verificar possíveis associações entre autorreferência de impacto vocal e os fatores relacionados ao uso de máscara facial. O nível de significância adotado foi de 5% (p < 0,05). Todas as análises foram realizadas utilizando o software JAMOVI, versão 2.3.

## RESULTADOS

Participaram do estudo 85 pessoas trans, com média de idade de 30,4 ± 9,31 anos, sendo 38 (44,7%) homens trans e 47 (55,3%) mulheres trans. A Tabela 1 apresenta os valores de idade e os escores do IDV-10 e do IFV distribuídos por gênero.

**Tabela 1:** Idade, escore no IDV-10 e no IFV de acordo com o gênero

	**Gênero**	**Média**	**Mediana**	**Desvio-padrão**	**Mínimo**	**Máximo**
Idade	Homem trans	28,0	27,0	7,54	19	54
	Mulher trans	32,3	32,0	10,19	18	57
IDV-10 total	Homem trans	12,2	11,5	7,81	0	27
	Mulher trans	14,5	14,0	8,29	0	37
IFV Fadiga e Restrição	Homem trans	13,9	13,5	7,74	0	30
	Mulher trans	16,4	15,0	9,62	0	35
IFV - Desconforto físico associado à voz	Homem trans	3,87	2,00	4,97	0	20
	Mulher trans	3,04	1,00	3,80	0	14
IFV - Recuperação com repouso vocal	Homem trans	6,18	8,00	4,42	0	12
	Mulher trans	6,87	8,00	4,56	0	12
IFV- Total	Homem trans	23,9	24,0	12,6	4	54
	Mulher trans	26,3	28,0	14,0	0	47

Análise estatística descritiva

Legenda: IDV-10 = Índice de Desvantagem Vocal 10; IFV = Índice de Fadiga Vocal

Conforme apresentado na Tabela 2 verificou-se que 56,5% dos participantes não se sentiram confortáveis com o uso das máscaras de proteção, enquanto 43,5% relataram conforto; 65,9% afirmaram não ter percebido alterações vocais durante a pandemia, enquanto 34,1% relataram alterações; e, por fim, 75,3% sentiram-se mais seguros socialmente com o uso da máscara, comparados aos 24,7% que não relataram essa sensação. A análise de correlação indicou relações significativas entre os escores do IDV-10 e os domínios do IFV, com exceção do domínio “Recuperação com repouso vocal” (ρ = 0,03; p = 0,744)

**Tabela 2:** Medidas de Ajustamento e coeficientes do modelo

**Modelo**	**Desviância**	**AIC**	**R^2^_CS_**	**R^2^_CS_**	**Teste ao Modelo Global**		
					**χ^2^**	**gl**	**p**
1	80,0	84,0	0,158	0,241	15,0	1	< ,001
Coeficientes do modelo – “Você se sentiu mais segura socialmente com o uso de máscaras de proteção”							
**Preditor**				**Estimativas**	**Erro-padrão**	**Z**	**p**
Intercepto				-0,423	0,295	-1,43	0,152
Você se sentiu confortável com o uso das máscaras de proteção?							
Sim – Não				-2,439	0,785	-3,11	0,002

Nota. As estimativas representam o Log das Chances de “Você se sentiu mais segura socialmente com o uso de máscaras de proteção = Não” vs. “Você se sentiu mais segura socialmente com o uso de máscaras de proteção = Sim”

Regressão logística binomial

Legenda: AIC = Critério de Informação de Akaike; R^2^_CS_ = coeficientes de determinação de Cox e Snell; χ^2^ = teste qui-quadrado; gl = graus de liberdade

A regressão logística binomial foi utilizada para investigar os preditores de “Você se sentiu mais segura socialmente com o uso de máscaras de proteção”. O modelo ajustado apresentou-se significativo com valores de R^2^ ajustado de Cox & Snell = 0,158 e R^2^ de Nagelkerke= 0,241), sugerindo que uma parte substancial da variabilidade foi explicada pela variável preditora: “Você se sentiu confortável com o uso das máscaras de proteção” mostrou-se estatisticamente significativo (Z = -3,11; p = 0,002).

Foram realizadas verificações de colinearidade, que apontaram valores ideais (VIF = 1,00; Tolerância = 1,00), assegurando a robustez do modelo. A análise de correlação entre o escore total do Índice de Desvantagem Vocal (IDV-10) e os domínios do Índice de Fadiga Vocal (IFV) demonstrou associações distintas entre os construtos avaliados. Observou-se correlação positiva moderada e estatisticamente significativa entre o IDV-10 e o domínio fadiga e restrição vocal do IFV (rho de Spearman = 0,530; p < 0,001), indicando que maiores níveis de desvantagem vocal estão associados a maior percepção de fadiga e limitação no uso da voz. Da mesma forma, identificou-se correlação positiva fraca, porém significativa, entre o IDV-10 e o domínio desconforto físico associado à voz (rho = 0,333; p = 0,002), sugerindo que o aumento da desvantagem vocal também se relaciona à maior presença de sintomas físicos vocais.

Por outro lado, não foi observada correlação estatisticamente significativa entre o escore total do IDV-10 e o domínio recuperação com repouso vocal do IFV (rho = 0,034; p = 0,759), indicando que a percepção de desvantagem vocal não se associa, neste grupo, à capacidade de recuperação vocal após períodos de repouso. Esses achados reforçam a convergência entre medidas de desvantagem vocal e fadiga vocal, especialmente nos aspectos relacionados à limitação funcional e ao desconforto físico.

## DISCUSSÃO

O presente estudo buscou compreender, por meio da autopercepção vocal de pessoas trans, como a utilização de máscaras de proteção facial influenciou o uso vocal e a interação social durante a pandemia de COVID-19. Segundo relato das participantes, a máscara serviu como forma de reduzir a ansiedade relacionada à voz, ao atenuar características que poderiam ser percebidas como discrepantes com a identidade de gênero. Assim, o conforto relatado não se restringe à proteção física contra o vírus, mas também reflete um efeito positivo na percepção social da identidade de gênero.

A voz representa um importante traço de identificação e comunicação do sujeito. Considerar como se qualifica a voz de pessoas trans é importante para compreender as demandas em relação ao bem-estar físico e psicossocial. Fatores como bem-estar social, psicológico e físico influenciam na qualidade de vida das pessoas^(7)^ e, consequentemente, na autopercepção vocal.

Diante dos estigmas vivenciados por pessoas trans, induzindo à intolerância que sofrem, denominada transfobia, a qualidade de vida torna-se um aspecto relevante a ser discutido. A transfobia é um fator que impacta diretamente a qualidade de vida das pessoas, contribuindo para o adoecimento.

Para contribuir com a melhora da qualidade de vida, é necessária a elaboração de ações de saúde voltadas à população trans, baseadas no conhecimento das demandas específicas^(7)^, no caso deste estudo, buscou-se conhecer as queixas pertinentes à voz e a relação com o uso de máscaras durante a pandemia por COVID-19.

A pandemia por SARS-COV 2 instituiu o uso de máscaras de proteção facial que, por sua vez, interferiu no uso da voz de toda a população e, especificamente, na voz das pessoas trans. A análise dos dados revelou que, embora algumas pessoas tenham se sentido desconfortáveis com o uso da máscara, a maioria relatou sensação de maior segurança social. Essa discrepância sugere que, mesmo diante de desconfortos físicos ou alterações vocais, um benefício percebido em termos de proteção contra a discriminação pode prevalecer. Os resultados da regressão logística reforçam essa interpretação, ao demonstrar que o conforto com a máscara é um preditor significativo da sensação de segurança social.

Conforme apontado por estudos anteriores, a voz é um elemento central na construção da identidade e na comunicação social^(7,8)^. No contexto das pessoas trans, a utilização da máscara pode funcionar como um recurso para atenuar aspectos vocais que, em determinadas situações, podem ser fonte de desconforto ou estigma. Tal porcentagem expressiva corrobora para que as máscaras de proteção promovem a segurança social, uma vez que ocultam parte da face, modificam o som da voz e, por fim, protegem a pessoa de ações transfóbicas. Este número indica a máscara como suporte na proteção à identidade, ao considerar a forte discriminação que atinge essa população^(9)^.

No contexto da autopercepção vocal, a máscara assume um papel ambíguo. Por um lado, ela atua como um mecanismo de defesa psicossocial, ao favorecer o que se denomina passabilidade de gênero. Esse conceito refere-se à forma como a pessoa é percebida em concordância com sua identidade de gênero e está associado ao conforto, à segurança social e à expressão de gênero^(8)^, reduzindo a exposição a situações de discriminação^(9)^. Por outro lado, ela pode ocasionar o abafamento do som, como demonstrado pelos valores de redução de até 3 a 4 dB para máscaras comuns e até 12 dB para máscaras N95^(10)^, o que implica em maior esforço vocal e potencial fadiga.

As pessoas trans buscam por uma voz que se adeque a sua identidade de gênero e, muitas vezes, a voz é um dos aspectos que mais interferem no processo de passabilidade^(11)^. Essa dualidade reforça a ideia de que, para pessoas trans, o uso da máscara vai além da proteção biológica e se configura como uma ferramenta de afirmação e segurança identitária.

Neste estudo, os valores correspondentes à fadiga vocal foram semelhantes entre homens e mulheres trans. Situação contrária foi encontrada em um estudo que constatou que mulheres cisgênero, por utilizarem mais a voz, autopercebem maior fadiga vocal que homens cisgênero^(12)^. Estudos comparativos em populações cisgênero demonstram, em alguns contextos, que mulheres podem relatar maior desvantagem vocal; entretanto, neste estudo, não foram observadas diferenças significativas entre homens trans e mulheres trans. Os processos de transição vivenciados por homens e mulheres trans apresentam similaridades, incluindo dimensões sociais, hormonais e, em alguns casos, cirúrgicas. Embora esses aspectos possam envolver objetivos específicos e distintos, eles compartilham elementos comuns que contribuem para a convergência dos percursos de transição, possivelmente em função das demandas psicossociais e do estresse envolvidos nessas experiências.

A análise das escalas utilizadas demonstrou correlações significativas entre o Índice de Desvantagem Vocal-10 (IDV-10) e os dois domínios do Índice de Fadiga Vocal (IFV), com exceção do domínio “Recuperação com repouso vocal”. Essa constatação indica que o aumento da percepção de desvantagem vocal está associado a maiores níveis de fadiga e desconforto físico, corroborando a necessidade de uma abordagem fonoaudiológica que contemple tanto os aspectos físicos quanto os psicossociais na intervenção com essa população.

Ademais, os dados indicaram que não houve diferenças significativas nos escores dos protocolos (IDV-10 e IFV) entre homens e mulheres trans, o que pode refletir os processos comuns de transição social e, em alguns casos, hormonal e cirúrgica vivenciados por esses grupos^(7)^. Esse achado sugere que, apesar das possíveis variações individuais, as demandas e desafios relacionados à produção vocal se apresentam de forma homogênea dentro da população trans, enfatizando a necessidade de estratégias de intervenção inclusivas e específicas. Tal resultado é intrigante, pois em outro estudo, com homens e mulheres cisgênero, também não houve associação estatística entre o IFV e o fator gênero^(11)^. Adicionalmente, investigações com populações cisgêneras sugerem que, apesar do IFV apresentar eficácia independente do gênero, diferenças na percepção de problemas vocais podem ser observadas, o que não foi observado no presente estudo.

Os dados evidenciam a urgência de ações fonoaudiológicas que abordem tanto os aspectos técnicos da produção vocal quanto as dimensões psicossociais, contribuindo para a melhoria da qualidade de vida e para a formulação de políticas públicas inclusivas. Os aspectos negativos na produção vocal apontados nos resultados sugerem a necessidade do atendimento fonoaudiológico acessível à população de acordo com os princípios da Universalidade e Integralidade do SUS, o que ainda não acontece^(13)^. A relevância desse estudo é reforçada pelo fato de que, embora o SUS reconheça a importância dos profissionais de Fonoaudiologia na equipe multidisciplinar de atenção à saúde das pessoas trans, tais profissionais ainda não constam na Portaria do Processo Transexualizador do Ministério da Saúde^(3)^.

Em virtude da qualidade vocal estar diretamente ligada à qualidade de vida, é válido ressaltar que um maior destaque a estudos como este, envolvendo a autopercepção vocal de pessoas trans e a pandemia da COVID-19, poderá servir como importante referência na atualização ou elaboração de novas Políticas Públicas para essa população^(7)^.

É reconhecida a importância de futuras pesquisas que incorporem critérios de exclusão mais específicos, como a delimitação de profissionais da voz, e que adotem desenhos longitudinais para elucidar, de forma mais robusta, os efeitos a longo prazo do uso de máscaras na autopercepção vocal e na segurança social de pessoas trans. Essa perspectiva ampliada pode contribuir para a formulação de estratégias preventivas e de intervenção mais eficazes, fortalecendo o papel da Fonoaudiologia no contexto da atenção integral à saúde dessa população.

Este estudo apresenta limitações, como o uso de uma amostra por conveniência, o que pode limitar a generalização dos resultados. Entretanto, este tipo de amostra se justifica pelo fato da população alvo, as pessoas trans, sofrerem invisibilizações nos censos populacionais o que também justifica a forma que foi conduzida a amostra. Para minimizar esta limitação, o trabalho buscou critérios de inclusão bem definidos e uma caracterização detalhada dos participantes para auxiliar a contextualizar os achados.

Outra limitação diz respeito à ausência de um grupo controle, o que poderia possibilitar comparações diretas entre pessoas trans e a população cisgênera quanto aos efeitos do uso de máscaras. Entretanto, esta escolha metodológica está alinhada ao objetivo principal do estudo, de compreender a experiência vocal e social de pessoas trans no contexto do uso de máscaras de proteção facial. A proposta não é estabelecer contrastes com outros grupos, mas dar visibilidade às vivências específicas dessa população, cuja realidade é marcada por atravessamentos identitários, sociais e comunicativos próprios.

## CONCLUSÃO

Foi possível identificar que o uso de máscaras de proteção facial exerce um duplo papel na experiência vocal e social de pessoas trans. Apesar do desconforto relatado por parte dos participantes, a sensação de segurança social foi significativamente elevada, indicando que os benefícios psicossociais podem superar os desafios físicos associados ao uso prolongado da máscara. Os resultados revelaram que o conforto com o uso da máscara é um preditor relevante para essa sensação de segurança, o que evidencia a importância desse fator no cotidiano da população estudada.

Adicionalmente, as correlações entre os escores do IDV-10 e os domínios do IFV sugerem que a percepção de desvantagem vocal está associada ao aumento da fadiga vocal e do desconforto físico. A ausência de diferenças estatísticas significativas entre homens e mulheres trans nos protocolos aplicados aponta para uma possível homogeneização das experiências vocais decorrente dos processos de transição social, hormonal e/ou cirúrgica.

Diante desses achados, destaca-se a urgência de desenvolver estratégias de intervenção fonoaudiológica que integrem aspectos técnicos da produção vocal a dimensões psicossociais, promovendo maior conforto, autenticidade vocal e bem-estar. Recomenda-se ainda a ampliação das pesquisas com amostras mais diversas e longitudinais, bem como a implementação de políticas públicas que considerem as especificidades da população trans no acesso à saúde vocal, à socialização segura e ao reconhecimento de suas identidades em contextos sociais diversos.
